# Attempted suicide of two confirmed SARS-CoV-2 infected patients in an isolation facility and recommendations to prevent COVID-19 suicides: a case report

**DOI:** 10.11604/pamj.2022.41.245.29660

**Published:** 2022-03-24

**Authors:** Benjamin Demah Nuertey, Kareem Mumuni, Joyce Addai, Sheba Kunfah, Rosemary Ivy Attibu, Daniel Acquah, Kwame Ekremet, Abdul-Rashid Haidallah, Ibrahim Baba Mahama, David Torgbor Adjah, Michael Damah, Braimah Baba Abubakari

**Affiliations:** 1 Public Health Department, Tamale Teaching Hospital, Northern Region, Tamale, Ghana,; 2 Community Health Department, University of Ghana Medical School, Korle-Bu, Accra, Ghana,; 3 Department of Obstetrics and Gynaecology, University of Ghana Medical School, Korle-Bu, Accra, Ghana,; 4 Department of Medicine, Korle-Bu Teaching Hospital, Korle-Bu, Accra,; 5 Clinical Psychology Unit, Tamale Teaching Hospital, Tamale, Ghana,; 6 Emergency Department, Tamale Teaching Hospital, Tamale, Ghana,; 7 Psychiatry Unit, Tamale Teaching Hospital, Tamale, Ghana,; 8 Tamale Teaching Hospital, Tamale, Ghana,; 9 Northern Regional Directorate, Ghana Health Service, Accra, Ghana

**Keywords:** Suicide, COVID-19, SARS-CoV-2, psychological, case report

## Abstract

COVID-19 pandemic has disrupted our way of life and continue to exert significant psychological impact. A surge in suicide has been associated with all previous major epidemics and pandemics. The suicide rate associated with COVID-19 pandemic would continue increasing if urgent measures are not put in place. We report two cases of attempted suicide among confirmed COVID-19 patients. The first case is a 30-year-old nurse who attempted suicide in an isolation facility and the second case is a 43-year-old male who travelled with his wife and a trusted friend from Burkina-Faso to Ghana to access haemodialysis care for his wife in a COVID-19 pandemic era. Unfortunately, the couple tested positive for SARS-CoV-2 infection. We discussed interventions to prevent suicide in treatment facilities. We recommend psychological assessment and counselling for all COVID-19 patients. We also recommend social interaction among patients in the isolation or treatment centres, and active management of COVID-19 related stigma and misinformation. Screening for means of suicide should be conducted in treatment facilities. Pre-test and post-test counselling are essential interventions. Also, telemedicine, telephone calls, computer assisted psychotherapy, mobile applications, self-guided digital interventions have been identified as effective tools for administering psychotherapeutic interventions to COVID-19 patients particularly in instances where face-to-face may not be possible.

## Introduction

Corona virus disease-19 (COVID-19) pandemic has disrupted several aspect of humanity´s normal ways of life and is associated with severe psychosocial impact including fears, worries, anxiety and suicide. With the extremely high infectivity rate and widespread social media reports of fatalities associated with its infection, fear of COVID-19 is a natural consequence. Most people in Ghana still consider severe acute respiratory syndrome corona virus-2 (SARS-CoV-2) infection a death sentence. Many individuals experience discrimination, stigma and in some circumstances ostracizing due to a diagnosis or community members suspicion of COVID-19. These has the potential to bring about psychological wellness issues among those with no past mental health challenges and possibly intensify the state of those with previous mental health issues [1]. Although there have been reliable data in relation to mental health correlates of the current pandemic such as insomnia, depression, post-traumatic stress disorder in places such as the UK, US and China, very little has been done about COVID-19 impact in Africa [1].

Burkina-Faso and Ghana recorded their first cases of COVID-19 on the 9^th^ March and 12^th^ March, 2020 respectively [2,3]. The government of both countries instituted containment measures such as social distancing, closure of borders, mandatory quarantine and use of masks [2,3]. As at mid-March, 2021, Ghana is ranked among the top 10 worst hit countries in Africa with over 88,000 confirmed case and over 700 deaths whilst Burkina Faso had over 12,400 confirmed cases and 145 deaths [4]. Data on general suicide rate showed that males had high age standardized suicide rates per 100,000 population compared to females in both Ghana (4.2 and 2.2) and Burkina-Faso (7.3 and 2.8) [5].

There are ample evidence that pandemics such as the 1918-1919 influenza pandemic, the 2003 severe acute respiratory syndrome (SARS) epidemic were all associated with increased suicide rate [6]. The story is not different in this COVID-19 pandemic. Some countries such as Bangladesh, Pakistan and Indian have already reported suicides associated with COVID-19 pandemic [7-9]. All of these supposedly COVID-19 suicides were among mostly non-COVID-19 patient who committed or attempted suicide due to the economic and social issues surrounding the COVID-19 pandemic. Majority of such case reports were data from press reports. Herein we present two cases of COVID-19 confirmed patients who attempted suicide in a treatment/isolation facility. Our interventions in managing the patient and recommendations to prevent suicide among SARS-CoV-2 infected patients are discussed.

## Patient and observation

**Case report 1:** case report one was a 30-year-old male, who is a nurse in a district hospital in Ghana. He was married and had a child. He had symptoms of fever, headache, nasal congestion and cough for which a test for malaria was carried out which confirmed malaria and malaria treatment was given. Symptoms were not subsiding and a test for COVID-19 was requested on the 23^rd^ of June 2020. The results came in positive for COVID-19 and he was detained at the isolation ward of the hospital in which he worked. He was not happy with that arrangement. He therefore escaped from isolation facility and went home. The police were informed, went to pick him up and brought him back to the isolation centre against his will. Security was beefed up to prevent further escape from isolation. While on admission, at the isolation centre, a health worker walked into his room to find him with a rope around his neck tied to the support of a ceiling fan in his room. He was standing on a table and was about to jump. He was rescued and a nurse was permanently stationed in his room until arrangements were made to refer him to a tertiary facility. An ambulance was sent to bring him to the company of paramedics and a doctor. He received clinical psychological assessment and counselling upon arrival to the infectious disease centre in Tamale Teaching Hospital (TTH) before admission. He spent 10 days on admission and had three extra counselling sections.

**Assessment outcome:** interview, psychological assessment and observation yielded him not having any past psychiatry history of relevance neither had there been any previous suicide attempt. He also did not present with any issue of delusions nor hallucinations with thought being content related. He generally maintained good eye contact and was oriented to place, person and time. Assessment of possible non-COVID related stressors did not yield any positive results with him engaging in what he considered 'normal' life's activities before his COVID diagnosis. He reported a subjective unit of distress in relations to events surrounding his COVID treatment as 8 (0-10 where 10 represented the highest form of distress). Feeding and sleep had been impacted prior to him being brought to this referral centre. He expressed sad affect with major expression of uncertainty in relation to going back to where he lived. The depression anxiety stress scale was also used to measure the dimensions of basic distress felt with him scoring 21 in depression, 24 in anxiety and 25 in stress. Even though there was thought intrusion, there was neither avoidance nor hyper arousal in relations to the police encounter. Themes identified within his possible contributors included; humiliation in relation to how he was handled: *"I felt humiliated by the forceful arrest and maltreatment by the police in the process of sending me to the isolation centre in the hospital where I work. I was arrested by the police in broad day light with several community members watching me as the police maltreated me and carried me to the isolation centre"*; also, there were feelings of worthlessness- *"I felt worthless and does not know how to face the community after treatment"*.

**Intervention and results:** psychoeducation on the possible psychosocial correlates of COVID especially in the area of anxiety, stigma and depression was given by the psychologist. Possible emphases were placed on disease novelty, infodemia and uncertainty which is making individuals hypervigilant and developing more protective approach of themselves therefore responding in inappropriate ways towards what they may consider as threats. Adherence counselling was also given. The target of this approach was to help prevent the previous non-adherence from re-occurring. Barriers to treatment such as confidentiality was identified and addressed in order to promote his compliance. Three additional sessions (cognitive therapy focused) were held with emphasis on identifying and managing possible thought patterns such as concern for family, life after recovery at where he lives that contribute to his negative emotional. This helped “retune” his mind to the treatment process and recovery and aided recovery. Psychoeducation and adherence helped alley his fears and also helped with his complying with the treatment that was given at this facility. After the third session, his subjective unit of distress has reduced to 3 whilst post-Depression Anxiety Stress Scales (DASS) scores were 12 for depression, 9 for anxiety and 12 for stress. After discharge, follow-up calls were carried out for up to six months from the treatment centre. Prominent complaint received was perceived stigma from the community members towards himself and wife even though wife never tested positive for SARS-CoV-2. He therefore applied for transfer out of the hospital where he currently works to a different facility due to the stigmatization.

**Case report 2:** the second case was a 43-year-old male resident in a community in Burkina Faso, 148 Km from the capital Ouagadougou, Burkina-Faso and 224 km from the Tamale Teaching Hospital (TTH) in Ghana. He, in the company of a trusted friend, accompanied his wife, who was diagnosed with end-stage renal failure to Ghana to access haemodialysis care. His trusted friend was more familiar to Ghana because of his frequent business travels. His first visit to Ghana was on 4^th^ of March where his wife was detained in a private medical facility located in the upper East Region of Ghana for two days and was subsequently referred to Tamale Teaching Hospital (TTH) for further care. Due to financial reasons, they waited for a week before reporting to TTH. He went to the Ghana- Burkina Faso boarder to meet a family relative who dispensed some cash loans to him for the management of his wife. After initial assessment of his wife on the day of presentation in TTH (12^th^ of March), an end stage renal failure diagnosis was made. He requested for discharge against medical advice on financial grounds and returned to his home town in Burkina Faso. While at home, the condition of the wife deteriorated and it took about two weeks to raise enough funds to return to Ghana for treatment in the company of the same friend. Even though by then Ghana was on partial lockdown, with land and air boarders being completely shut, they still managed to use unapproved land boarder route into Ghana. Inside Ghana, public transport was used as main means of movement. His wife had her first successful dialysis on the 27^th^ of March. With closure of the land boarder, the protocols demanded the three undergo mandatory quarantine and testing for COVID-19. The couple was kept in one room whilst the friend was kept in another room at a hotel repurposed to serve as a quarantine, isolation and treatment centre for asymptomatic and mild COVID-19 cases. The results received on the 29^th^ of March indicated the couple was positive whilst their friend tested negative. These results were disclosed in group with no special pre or post-test counselling given. After the results, he started refusing meals under the pretext of either not being hungry or not liking Ghanaian foods. He was also seen to be more withdrawn. The night of the third day, his wife woke up in the middle of the night to see her husband with a cloth tired around his neck and was attempting to tie it up on a support. She rushed out of the room to inform their friend who came in to rescue him from the process.

**Assessment outcome:** there was significant language barrier as he and his wife did not speak nor were they able to write English or French. Assessment was done through a tribe mate living and working in Ghana who served as an interpreter. Psychiatry and psychological evaluations were done by the psychiatry clinic and the psychologist. Results indicated that he was not exhibiting any psychotic symptom with areas of perception, thought and speech being considered normal. Significant area impacted was mood which was generally expressed as sadness. He also had high level uncertainty and confusion in relation to reason being isolated. Assessment of pre-COVID situation indicated significant financial stress and difficulty handling wife's condition. He had however not had any history of mental illness nor suicide attempt. There was no family history of mental illness. He reported a subjective unit of distress in relations to events surrounding his COVID treatment as 9 on a scale of 0-10 where 10 represented the highest form of distress. This distress according to him, mainly emanated from fear of being killed by the Ghanaians. *"I suspect the Ghanaian health officials want to kill us, I will kill myself before they succeed in killing us"*.

**Intervention and results:** primary focus of psychological intervention was to help instil some level of safe, reliable and comfortable support systems around him which would help reduce his fear of being targeted on a “foreign” land. To achieve this, two areas of support were identified; family and friends and primary caregivers. With his consent, well-meaning leaders from his tribe living and working in Ghana were brought in to pay occasional visits and to also ascertain level of subjective treatment progress. These individuals were coached on issues such as assuring confidentiality. The caregivers also acted as friends by helping him reconnect with his friend, through phone calls even though he initially had no phone. Other tangible supports such as items for self-care for himself and wife were also provided to help possibly increase the appraisal of support as positive rather than detrimental. Because the situation of the wife was also a prominent stressor, even though admission and related activities for COVID-19 care were free to him and his wife, additionally, all dialysis services, laboratory and radiological investigations and medications for co-morbid conditions of his wife were administered at no cost. This was aimed at reducing the possible stress that might be related to the wife's underlying condition. In all, four therapy sessions were planned with emphasis on highlighting the possible support systems available, focusing on the positives in the recovery process and relaxation training (guided imagery). After the fourth session, he reported subjective unit of distress as 3 with significant contributors being improved relationship with staff and improvement in health of his wife.

Medically, he completed five days of 500mg tablet azithromycin, ten days of tablet hydroxychloroquine 200mg three times daily and daily dose of a multivitamin tablet containing among other vitamins and minerals; Zinc and vitamin C. He obtained two negative follow up results for SARS-CoV-2 reverse transcription polymerase chain reaction (RT-PCR) and was discharged on the 16^th^ March 2020. He left with his wife to Burkina-Faso on the 18^th^ of March 2020. Last follow-up call was middle of October 2020 and he was in good health.

## Discussion

These case presentations highlight suicidal attempts of COVID-19 patients in isolation facilities, which were foiled by timely intervention. Suicide is considered the ultimate human sacrifice in circumstances when one can no longer bear the mental pressure and suffering [10]. Several stressors may trigger suicide in a predisposed individual. With regards to case number 1, police arrest in Africa, which was carried out in broad daylight with several community people watching the arrest, is enough in certain individuals to trigger suicidal ideation. With regards to case report two, moving out of your country to seek medical care for a loved one with end-stage kidney failure, mandatorily being quarantine, diagnosis of COVID-19, coupled with economic and social problems could adversely affect mental health including depression, anxiety, and stress. It is well established that, socio-economic as well as pandemic related issues can trigger psychological mediators such as sadness, worry fear, anger, guilt, helplessness, loneliness, and nervousness [10]. Also, studies have shown that, infectious disease pandemics such as that of COVID-19 can amplify the negative effects of psychological reactions such as hypochondriasis, anxiety and overall quality of the individual health and wellbeing [11]. We believe, that a complex set of economic, social, medical circumstance might have triggered suicidal attempts in the cases presented above.

The news of having COVID-19 can trigger suicidal ideation in some vulnerable persons. Many across Africa during the onset of the pandemic believe that having SARS-CoV-2 infection is a death sentence. There were widely circulated myths on social media in Africa that, China and other badly affected COVID-19 countries actively implement and cover up mass killings of the infected as a way of controlling the spread of the virus. Some of these social media myths include vivid videos of human suffering and killings as purported to be on-going in countries badly hit by SARS-CoV-2 infection [12]. Most patients are still afraid of isolation centres because of this believe that, they would be killed in these isolation centre. These myths could have contributed to the heightened fear of COVID-19 and the suicidal ideation in suicidal behaviors (SB). Such fears are best controlled with adequate pre-test and post-test counselling.

However, pre-test and post-test counselling for the first patient, the second and his wife were not adequately conducted. Pre-test and post-test counselling is best carried out on a one-on-one basis. This allows the individual to ask all questions which otherwise might be scared to ask in a group. Hence, adequate pre and post-test counselling are necessary to avoid COVID-19 related suicides in an era where social distancing and sophisticated personal protective equipment lessen the effectiveness of psychological assessment and counselling. Psychological assessment and counselling are essential for all COVID-19 patients. Pandemics have the potential of worsening pre-existing mental illness. Pandemic associated fear, isolation and physical distancing could also exacerbate mental illness [13]. Patients with existing mental health illness are more vulnerable during pandemics. There is therefore the need to assess, pick up and treat all infected individuals early in their management to avoid exacerbation of mental illness, which could lead to, self-harm. Telemedicine, telephone consultations are equally effective when face to face approach are not possible [14]. Studies have shown that telemedicine is suitable for psychiatric treatment with no loss of effectiveness when compared to face-to face therapy [14,15]. Other easy access technologies such as computer assisted psychotherapy, mobile applications, self-guided digital interventions and telephone calls have been found to be equally effective in times such as pandemics [16-18] where social distancing and isolation is the norm.

Social isolation, entrapment, loneliness are recognized factors that increase the risk of suicides [13,19]. These may be prominent in isolation or treatment facilities where inmates are confined to their rooms. It is encouraged that, among patients in the treatment centres for COVID-19 or future epidemics, patients should be allowed the social interaction among patients when possible. This has the potential to speed up the healing process. From our experience in a treatment centre, and anecdotal evidence suggests that, patients that do confine themselves to their rooms have slower recovering rate for COVID-19 compared to those who move out to common areas and are involved in interactions. Also, those who remain confined to their rooms were moodier and more depressed looking compared to those who move out of their rooms. We therefore recommend social interactions among the socially distanced patients who are at the treatment centre as a means of improving mental health and accelerating recovery.

Also, treatment or isolation facilities must ensure that, access to means of suicide is not available in the treatment centres. Access to means is a major risk factor for suicides. COVID-19 pandemic in most part of Africa has been approached with both facility and home-based management depending on set criteria. Irrespective of the choice of management approach, healthcare providers must ensure that, the care environment is as much as possible devoid of certain lethal means such as pesticides, analgesics, firearms and sharps. Other intervention areas to explore by healthcare personnel include encouraging health promoting behaviours, managing COVID-19 related stigma and misinformation, integration of medical and psychological care for COVID-19 patients and empowering patients and their families [10,20] to stay healthy in this pandemic times.

## Conclusion

We presented two cases of attempted suicide in a COVID-19 treatment facility. Suicide is considered the ultimate human sacrifice in circumstances when one can no longer bear the mental pressure and suffering. Several stressors may trigger suicidal ideation in some vulnerable persons. Psychological assessment and counselling are essential for all COVID-19 patients to identify patients who may have underlying predisposition for suicide. We also recommend social interaction among patients in the isolation or treatment centres, and active management of COVID-19 related stigma and misinformation. Screening for means of suicide should be conducted in treatment facilities. Also, pre-test and post-test counselling; face-to-face, telemedicine, telephone, computer assisted psychotherapy, mobile applications, self-guided digital interventions are effective tools for administering interventions to COVID-19 patients.
